# PROFILE OF PATIENTS WITH OSTEOPOROTIC FRACTURES AT A TERTIARY ORTHOPEDIC TRAUMA CENTER

**DOI:** 10.1590/1413-785220182602185325

**Published:** 2018

**Authors:** MICHAEL MINSU SHU, ANDRE LANGES CANHOS, GUILHERME PEREIRA OCAMPOS, PEROLA GRIMBERG PLAPLER, OLAVO PIRES CAMARGO, MARCIA UCHOA DE REZENDE

**Affiliations:** 1. Osteometabolic Diseases Group, Instituto de Ortopedia e Traumatologia, Hospital das Clinicas HCFMUSP, Faculdade de Medicina, Universidade de São Paulo, SP, Brazil.; 2. Department of Orthopedics and Traumatology, Faculdade de Medicina, Universidade de São Paulo, SP, Brazil.

**Keywords:** Osteoporotic fractures, Osteoporosis, Epidemiology, Diagnosis, Bone density, Prevalence., Fraturas por osteoporose, Osteoporose, Epidemiologia, Diagnóstico, Densidade óssea, Prevalência

## Abstract

**Objective:**

To evaluate the profile of patients with osteoporotic fractures treated at a tertiary orthopedic hospital.

**Methods:**

Using questionnaires, 70 patients with osteoporotic fractures (OF) were compared with 50 outpatients with multiple osteoarthritis (OA) followed through an outpatient clinic.

**Results:**

The OF group was older (p <0.001), less heavy (p=0.003), had lower BMI (p=0.006), was more likely to be white (p=0.011), was less likely to be married (p=0.008), and had previous falls, previous fractures, old fractures (>1 year), falls in the last 12 months, fractures due to falls, and needed more assistance (p<0.05). They also had lower Lawton & Brody Instrumental Activities of Daily Living scores (p <0.05) and reported less lower limb disability, foot pathology, muscle weakness, hypothyroidism, and vitamin D intake than patients in the OA group. White race, previous falls, and previous fractures increase the risk of osteoporotic fractures by 10.5, 11.4, and 4.1 times, respectively. The chance of fracture dropped 29% for each one-unit increase in Lawton & Brody IADL score. Married participants had fewer fractures than participants with other marital status.

**Conclusion:**

Together, race, marital status, previous falls, foot pathologies, previous fractures, and IADL scores define the profile of patients with osteoporotic fractures. Level of Evidence III; Case control study.

## RESUMO

**Objetivo:**

Avaliar o perfil dos pacientes com fraturas osteoporóticas atendidos em hospital de atendimento terciário ortopédico.

**Métodos:**

Setenta pacientes com fraturas osteoporóticas (FO) foram comparados a 50 pacientes com acompanhamento ambulatorial de osteoartrite (OA) por meio de questionários.

**Resultados:**

O grupo FO apresentou média de idade maior (p < 0,001), menor peso (p = 0,003), menor IMC (p = 0,006), maior frequência de pacientes brancos (p = 0,011), menor frequência de casados (p = 0,008), mais quedas prévias, fraturas prévias, fratura antiga (> 1 ano), queda nos últimos 12 meses, fratura por causa da queda e necessitam de mais auxílio (p < 0,05); menor Lawton e Brody AIVD (Atividades instrumentais da vida diária, p < 0,05), reportando menos deficiência de membros inferiores, patologia nos pés, fraqueza muscular, hipotireoidismo e consumo de vitamina D do que pacientes do grupo OA. Raça branca, quedas e fraturas prévias aumentam o risco de fraturas osteoporóticas em 10,5, 11,4 e 4,1 vezes respectivamente. A chance de fratura foi reduzida em 29% a cada aumento de uma unidade no Lawton e Brody AIVD. Casados fraturam menos que outros estados civis.

**Conclusão:**

Conjuntamente, a raça, estado civil, quedas prévias, patologias nos pés, fraturas prévias e AIVD definem o perfil dos pacientes com fraturas osteoporóticas deste centro. Nível de Evidência III; Estudo de caso-controle.

## INTRODUCTION

Osteoporosis is a chronic disease characterized by progressive reduction of bone mass, leading to decreased bone strength and greater risk of fractures;^1^ it is considered a public health problem worldwide. It has been estimated that 9 million osteoporotic fractures occur each year, the equivalent of one fracture every 3.5 seconds.^2^ Although this is the most common bone disease,^3^ many patients are not treated until the first fracture occurs. The Brazilian population is in the process of aging, as can be seen in the epidemiologic pyramids for the years 2017 and 2050.^4^ This aging is accompanied by an increase in the prevalence of osteoporosis and the incidence of falls and fractures.^5^ These fractures are associated with increased mortality, decreased functional capacity and quality of life,^6^ ^-^ ^9^ and increased spending in the health system. It is estimated that approximately 50% of women and 20% of men 50 years of age or over will suffer an osteoporotic fracture during their lives.

Even though osteoporosis and osteopenia are a growing problem in older people, attempts to analyze the characteristics of osteoporotic patients in Brazil are rare.

The objective of this study was to evaluate the epidemiological profile of the population affected by osteoporotic fractures (fractures of the proximal femur, the proximal humerus, the distal radius, and the thoraco-lumbar spine) treated in a tertiary orthopedic hospital over a three-month period, with or without a previous diagnosis of osteopenia or osteoporosis, in an attempt to correlate the clinical characteristics present in patients treated for osteoarthritis during the same period.

Primary objective: To explore the epidemiological profile of patients with osteoporotic fractures treated in a tertiary orthopedic hospital, identifying factors potentially related to this fracture in relation to patients treated for osteoarthritis during the same period.

Secondary objective: To describe the types of osteoporotic fractures treated in a tertiary center, along with function and bone mineral density in these patients.

## MATERIALS AND METHODS

This study was conducted at the Osteo-Metabolic Diseases Group at the Instituto de Ortopedia e Traumatologia do Hospital das Clínicas da Faculdade de Medicina da Universidade de São Paulo (IOT-HC-FMUSP) with the approval of the institutional review board (number 76629217.3/0000.0068).

All participants were patients with osteoporotic fractures treated over a three-month period in 2017 and patients with osteoarthritis of the knee (of this group, only those treated in the osteometabolic disease group at a tertiary orthopedic hospital).

Inclusion criteria: Study group (osteoporotic fractures, OF): Patients above 45 years of age presenting any one or a combination of the following fractures: proximal femur, proximal humerus, distal radius, and thoraco-lumbar spine, with a mechanism of low-energy trauma. Patients with high-energy fractures were not included.

Control group (patients with osteoarthritis, OA): Patients above 45 years with clinical/radiographic diagnosis of osteoarthritis of the knee,^10^ isolated or not, with and without comorbidities.

Exclusion criteria: Age below 45 years; suspicion or confirmation of pathological fractures; patient unwilling to participate.

### Interventions

The participants filled out a questionnaire (Table 1) collecting data on demographic profile, fracture type, race, patient level of education, habits, personal history, previous fractures, level of physical activity, aids for locomotion, place and time of the accident which caused the fracture, use of medications and behavioral measures to treat osteoporosis, and functional assessment [Katz and Lawton and Brody].^11^ ^,^ ^12^ Patients with proximal femur fracture completed the Harris Hip Score (HSS)^13^ and fragility score (SHARE) questionnaires.

**Table 1:** Evaluation of post-osteoporotic fracture patients and controls.

Identification	
Age	
Sex	Male: 0 / Fem: 1
Weight	
Height	
Race	White: 0 / Nonwhite: 1
Marital status:	Married: 0 / Widowed: 1 / Single: 3 / Other = 4
Lives with	Number of people
Kinship	Alone: 0 / Companion: 1 / Child: 2 / Grandchild: 3 / Other: 4
Education	Illiterate: 0 / Literate: 1
**Number of years of school**	
Father or mother with hip fracture?	No: 0 / Yes: 1
Current smoker?	No: 0 / Yes: 1
Glucocorticoids	No: 0 / Yes: 1
Rheumatoid arthritis?	No: 0 / Yes: 1
Secondary osteoporosis?	No: 0 / Yes: 1
Alcohol: >3 drinks per day?	No: 0 / Yes: 1
Sedatives?	No: 0 / Yes: 1
Previous falls?	No: 0 / Yes: 1
Cognitive deficit?	No: 0 / Yes: 1
Visual impairment?	No: 0 / Yes: 1
Disability of lower limbs?	No: 0 / Yes: 1
Foot pathology?	No: 0 / Yes: 1
Change in balance?	No: 0 / Yes: 1
Muscle weakness?	No: 0 / Yes: 1
Changes in gait?	No: 0 / Yes: 1
Postural hypotension?	No: 0 / Yes: 1
Dizziness?	No: 0 / Yes: 1
Depression/Apathy/Confusion?	No: 0 / Yes: 1
Diabetes?	No: 0 / Yes: 1
HBP?	No: 0 / Yes: 1
Hypothyroidism?	No: 0 / Yes: 1
Previous fractures?	No: 0 / Yes: 1
Old fracture (> 1 year)?	No: 0 / Yes: 1
Current fracture?	No: 0 / Yes: 1
**Date of current fracture?**	
Fractured limb	Spine: 0 / Lumbar Spine: 1 / R Hip: 2 / L Hip: 3 / R Wrist: 4 / L Wrist: 5 / R Shoulder: 6 / L Shoulder: 7
Physical activity before fracture?	No: 0 / Yes: 1
Type of activity	Weight training: 0 / Stretching 1 / Water or pool exercise: 2 / Walking: 3 / Cycling: 4
Physical activity after fracture?	No: 0 / Yes: 1
Type of activity	Weight training: 0 / Stretching 1 / Water or pool exercise: 2 / Walking: 3 / Cycling: 4
Frequency	1x month: 0 / 2X month: 1 / 3X month: 2 / 1X week: 3 / 2X week: 4 / 3X week: 5 / >4X week: 6 / Never: 7
Fear of falling?	No: 0 / Yes: 1
Fall in last 12 months?	No: 0 / Yes: 1
**Number of falls?**	
Where?	At home: 0 / Outside the home: 1
Factors	Dizziness: 0 / Tripped: 1 / Slipped: 2 / Weakness or lower limb instability: 3 / Other: 4
Fracture from fall?	No: 0 / Yes: 1
Assistance	Cane: 0 / Crutches: 1 / Walker: 2 / Wheelchair: 3 / None: 4
Mechanism of trauma?	Fall from height: 0 / Same-level fall: 1 / Direct trauma: 2 / Twisting: 3 / Carrying weight: 4
Time of Accident	7:00 -11:00: 0 / 11:01 - 15:00 1 / 15:01 - 19:00 2 / 19:01 - 22:00 3 / 22:01 - 7:00 4
Previous conduct related to current fracture?	Analgesic medication: 0 / Cast or vest: 1 / Surgery: 2 / Physical therapy: 3
Prior diagnosis of osteoporosis?	No: 0 / Yes: 1
Calcium supplementation?	No: 0 / Yes: 1
Sun exposure 3x week?	No: 0 / Yes: 1
Vitamin D supplementation?	No: 0 / Yes: 1
**If yes, how many IU?**	
Taking medication for osteoporosis?	No: 0 / Yes: 1
Katz ADL	No: 0 / Yes: 1 - (Maximum: 6)
Lawton & Brody IADL	No: 0 / Yes: 1 - (Maximum: 8)

### Statistical analysis

Patient characteristics were described using absolute and relative frequencies according to groups for the qualitative variables, and association was verified using the chi-square or Fisher’s exact tests. Summary measures (mean and standard deviation or median, minimum, and maximum) were calculated according to groups for quantitative variables and the groups were compared using Student’s t-test or the Mann-Whitney test.

The unadjusted odds ratio was estimated for each variable to approximate the chance of osteoporosis with the respective intervals, with 95% confidence.

The multiple logistic regression model was used to explain the osteoporosis group, selecting the variables that showed statistical significance in the bivariate tests and using backward stepwise selection with a 5% criterion for entry and exit of the variables (p<0.05).

IBM SPSS for Windows software version 20.0 was used for these analyses, and Microsoft Excel 2003 was used to tabulate the data. The tests were performed at a 5% significance level.

## RESULTS

The results of the questionnaires applied to 70 patients with osteoporotic fractures (OF) and 50 patients with osteoarthritis (OA) of the knee (or osteoarthritis of multiple joints including the knee) are summarized in Tables 2-4.

**Table 2:** Description of characteristics present in both groups and the results of unadjusted analyses.

	Group						
Variable	Control	Osteoporosis	Total	OR	IC (95%)		p
	(N = 50)	(N = 70)	(N = 120)		Below	Above	
Sex (female), n (%)	39 (78)	49 (70)	88 (73.3)	0.66	0.28	1.53	0.329
Age (years), mean ± SD	66.7 ± 9.6	75.1 ± 11.7	71.6 ± 11.6	1.07	1.03	1.12	<0.001*
Weight (Kg), mean ± SD	72.9 ± 11.2	66 ± 13.1	68.8 ± 12.8	0.96	0.92	0.99	0.003**
Height (cm), mean ± SD	162.3 ± 7.3	161.4 ± 8.8	161.8 ± 8.2	0.99	0.94	1.04	0.601**
BMI (Kg/m²), mean ± SD	27.9 ± 4.3	25.3 ± 5.1	26.3 ± 5	0.89	0.82	0.97	0.006**
Education (years of school), median (min.; max.)	8 (0; 30)	8 (0; 18)	8 (0; 30)	0.98	0.91	1.05	0.648£
Race (White), n (%)	35 (70)	62 (88.6)	97 (80.8)	3.32	1.28	8.61	0.011
Marital status, n (%)							0.008
Married	33 (66)	25 (35.7)	58 (48.3)	1.00			
Widowed	7 (14)	22 (31.4)	29 (24.2)	4.15	1.53	11.24	
Single	3 (6)	11 (15.7)	14 (11.7)	4.84	1.22	19.21	
Other	7 (14)	12 (17.1)	19 (15.8)	2.26	0.78	6.58	
Lives with, median (min.; max.)	1 (0; 3)	1 (0; 6)	1 (0; 6)	1.301	0.95	1.78	0.370£
Father or mother with hip fracture, n (%)	3 (6)	4 (5.7)	7 (5.8)	0.95	0.20	4.44	>0.999*
Current smoker, n (%)	6 (12)	8 (11.4)	14 (11.7)	0.95	0.31	2.92	0.923
Glucocorticoids, n (%)	3 (6)	5 (7.1)	8 (6.7)	1.21	0.27	5.29	>0.999*
Rheumatoid arthritis, n (%)	0 (0)	2 (2.9)	9 (7.5)	2.71	0.04	0.91	0.751
Secondary osteoporosis, n (%)	7 (14)	4 (5.7)	11 (9.2)	0.37	0.10	1.35	0.198*
Alcohol: >3 drinks per day, n (%)	3 (6)	2 (2.9)	5 (4.2)	0.46	0.07	2.87	0.648*
Sedatives, n (%)	7 (14)	9 (12.9)	16 (13.3)	0.91	0.31	2.62	0.856
Previous falls, n (%)	13 (26)	38 (54.3)	51 (42.5)	3.38	1.54	7.43	0.002
Cognitive deficit, n (%)	3 (6)	9 (12.9)	12 (10)	2.31	0.59	9.01	0.217
Visual impairment, n (%)	25 (50)	30 (42.9)	55 (45.8)	0.75	0.36	1.56	0.439
Disability in lower limbs, n (%)	13 (26)	7 (10)	20 (16.7)	0.32	0.12	0.86	0.020
Foot pathology, n (%)	18 (36)	6 (8.6)	24 (20)	0.17	0.06	0.46	<0.001
Changes in balance, n (%)	19 (38)	25 (35.7)	44 (36.7)	0.91	0.43	1.92	0.798
Muscle weakness, n (%)	24 (48)	21 (30)	45 (37.5)	0.46	0.22	0.99	0.045
Changes in gait, n (%)	24 (48)	24 (34.3)	48 (40)	0.57	0.27	1.19	0.131
Postural hypotension, n (%)	10 (20)	13 (18.6)	23 (19.2)	0.91	0.36	2.29	0.845
Dizziness, n (%)	13 (26)	18 (25.7)	31 (25.8)	0.99	0.43	2.26	0.972
Depression/Apathy/Confusion, n (%)	11 (22)	17 (24.3)	28 (23.3)	1.14	0.48	2.70	0.770
Diabetes, n (%)	19 (38)	22 (31.4)	41 (34.2)	0.75	0.35	1.60	0.454
HBP, n (%)	30 (60)	35 (50)	65 (54.2)	0.67	0.32	1.39	0.278
Hyperthyroidism, n (%)	14 (28)	8 (11.4)	22 (18.3)	0.33	0.13	0.87	0.021
Previous fractures, n (%)	8 (16)	35 (50)	43 (35.8)	5.25	2.16	12.78	<0.001
Old fracture (> 1 year), n (%)	8 (16)	35 (50)	43 (35.8)	5.25	2.16	12.78	<0.001
Physical activity before fracture, n (%)	19 (38)	21 (30)	40 (33.3)	0.70	0.33	1.51	0.359
Fear of falling, n (%)	34 (68)	44 (62.9)	78 (65)	0.80	0.37	1.72	0.560
Fall in last 12 months, n (%)	17 (34)	41 (58.6)	58 (48.3)	2.74	1.29	5.83	0.008
Fracture from fall, n (%)	2 (4)	67 (95.7)	69 (57.5)	536.00	86.23	3331.95	<0.001
Assistance, n (%)	5 (10)	35 (50)	40 (33.3)	9.00	3.19	25.36	<0.001
Prior diagnosis of osteoporosis, n (%)	14 (28)	26 (37.1)	40 (33.3)	1.52	0.69	3.33	0.295
Calcium supplementation, n (%)	14 (28)	20 (28.6)	34 (28.3)	1.03	0.46	2.30	0.945
Sun exposure 3x week, n (%)	24 (48)	36 (51.4)	60 (50)	1.15	0.56	2.37	0.711
Vitamin D supplementation, n (%)	28 (56)	22 (31.4)	50 (41.7)	0.36	0.17	0.76	0.007
Taking medication for osteoporosis, n (%)	4 (8)	8 (11.4)	12 (10)	1.48	0.42	5.23	0.537
Katz ADL, median (min.; max.)	6 (2; 6)	6 (1; 6)	6 (1; 6)	0.73	0.46	1.16	0.090£
Lawton & Brody IADL, median (min.; max.)	8 (1; 8)	7.5 (0; 8)	8 (0; 8)	0.83	0.70	0.99	0.015£

Chi-square test; * Fisher's exact test; ** Student's t-test; £ Mann-Whitney test.

**Table 3:** Result of the joint model describing the osteoporosis group according to evaluated variables.

Variable	OR	IC (95%)		p
		Below	Above	
Race (White)	10.48	1.61	68.20	0.014
Marital status				
Married	1.00			
Widowed	4.93	0.94	25.99	0.060
Single	57.15	2.81	1162.39	0.008
Other	10.85	1.80	65.56	0.009
Previous falls	11.39	2.18	59.45	0.004
Foot pathologies	0.13	0.02	0.74	0.022
Muscle weakness	0.15	0.03	0.77	0.024
Hypothyroidism	0.14	0.03	0.75	0.022
Previous fractures	4.13	1.12	15.23	0.033
Katz & Lawton IADL	0.71	0.53	0.95	0.020

Multiple logistic regression.

**Table 4:** Description of characteristics that were evaluated only in patients with osteoporosis.

Variable	Description
Fractured limb, n (%)	
Lumbar Spine	2 (2.9)
Hip	57 (81.4)
Wrist	4 (5.7)
Shoulder	7 (10)
Physical activity after fracture, n (%)	
No	47 (67.1)
Yes	23 (32.9)
Ca Supplementation, n (%)	
No	35 (71.4)
Yes	14 (28.6)
SHARE FI exhaustion, n (%)	
No	29 (58)
Yes	21 (42)
SHARE FI Appetite, n (%)	
Reduced	8 (16)
Maintained	37 (74)
Increased	5 (10)
HSS Pain	
mean ± SD	32.2 ± 12.6
median (min.; max.)	40 (8; 44)
HSS Function	
mean ± SD	26.3 ± 12
median (min.; max.)	27.5 (0; 47)
HSS ADM	
mean ± SD	2.4 ± 0.8
median (min.; max.)	2.2 (0.9; 4)
HSS ADM Deformity	
mean ± SD	3.3 ± 1.3
median (min.; max.)	4 (1; 4)
HSS Total	
mean ± SD	65.1 ± 19.6
median (min.; max.)	72 (20.9; 97)
DMO COL T-Score	
mean ± SD	-2 ± 1.8
median (min.; max.)	-2.1 (-4.8; 2.6)
DMO FN T-Score	
mean ± SD	-2.7 ± 0.6
median (min.; max.)	-2.6 (-3.7; -1.7)
DMO TH T-Score	
mean ± SD	-2.5 ± 0.8
median (min.; max.)	-2.8 (-3.8; -1.3)
DMO Troc T-Score	
mean ± SD	-2.4 ± 0.7
median (min.; max.)	-2.4 (-2.9; -1.9)

Although there were 70 patients with osteoporosis, some information was missing for all variables.

Table 2 shows that in isolation, patients with osteoporosis were statistically older on average (p<0.001), were less heavy and had lower BMI (p=0.003 and p=0.006, respectively), the frequency of white race was statistically higher in patients with osteoporosis (p=0.011), patients with osteoporotic fractures were statistically less likely to be married (p=0.008), and this group had more previous falls, previous fractures, old fractures (> 1 year), falls over the past 12 months, fractures from falls, and needed more assistance (p<0.05) than patients with OA. Patients with osteoporotic fractures reported less disability in the lower limbs, pathology in the feet, muscle weakness, hypothyroidism, and vitamin D consumption than patients with OA. Using the functional scale by Lawton and Brody,^12^ their scores for instrumental activities of daily living (IADL) were lower (p<0.05).

Table 3 shows that together, race, marital status, previous falls, pathologies in the feet, muscle weakness, hypothyroidism, previous fractures, and Lawton and Brody IADL score^12^ explained the patients with osteoporosis independent of the other characteristics we assessed (p<0.05). White patients were 10.48 times more likely to present osteoporosis than nonwhite patients, single patients and those with other marital status had a statistically greater chance of osteoporosis than married patients, patients who had previous falls were 11.39 times more likely to have osteoporosis than patients without previous falls, and patients with previous fractures were 4.13 times more likely to have osteoporosis than patients without previous fractures. Pathologies of the feet, muscle weakness, and hypothyroidism presented similar protections for osteoporosis, with the chance of osteoporosis approximately 86% less for each of these characteristics, and each one-unit increase in the Katz and Lawton IADL score^12^ decreased the chance of osteoporosis by 29%.

Table 4 shows the profile of patients with osteoporotic fractures treated in a tertiary trauma center, with an 81% incidence of patients with hip fractures, confirming that osteoporosis accompanies this fracture in mean bone densitometry values.

## DISCUSSION

Osteoporosis is a chronic disease characterized by progressive decrease in bone mass, leading to decreased bone strength and greater risk of fractures.^1^ This disease can be characterized as primary or secondary. Primary osteoporosis can occur in both sexes at any age, but often occurs after menopause in women and later in men.^1^

In this study we observed that the patients with osteoporotic fractures were older, a greater number were women (similar to the group with OA), weighed less, had lower BMI, and whites were more prevalent (Table 2), consistent with findings in other studies.^1^ ^,^ ^14^ ^,^ ^15^ Perhaps because of the size and characteristics of the sample (older adults, Caucasians, and hip fractures were more prevalent) (Tables 2, 3 and 4), consumption of glucocorticoids, and consumption alcohol and tobacco were not seen to have a large influence, as described in the literature,^1^ ^,^ ^14^ ^-^ ^16^ but we found a protective relationship against osteoporotic fractures in married patients in relation to those with other marital status. (Tables 2 and 3) Pluskiewicz et al.^16^ reported a tendency for more fractures in widows.

Patients with osteoporosis presented more previous falls and more falls in the past 12 months, which together with the bone fragility caused by osteoporosis explains the higher incidence of fractures resulting from falls, old fractures (>1 year), and previous fractures. The higher number of falls can be partially explained by greater age and occasional sarcopenia in the OF group,^1^ ^,^ ^15^ ^,^ ^17^ although these patients reported less disability of the lower limbs, feet pathologies, and muscle weakness than younger patients with OA. (Tables 2 and 3) This could be partially explained by patients with OA who receive outpatient care for arthritis of the knee (isolated or involving multiple joints) which includes an educational program and periodic evaluations of functionality, raising awareness among these patients of the functional loss and deformities they exhibit.^18^ ^,^ ^19^ This differs from the group receiving care for fracture, who still need to be assessed functionally and complete an educational program to develop awareness of what led to the osteoporotic fracture, the types of osteoporosis, the risks of their condition, and necessary treatment, along with consolidation of the fracture in question. Because a significant number of patients in the OF group did not report muscle weakness, muscle weakness was statistically indicated as a “protective factor” against osteoporotic fractures. (Tables 2 and 3) Muscle weakness was not assessed objectively. We believe that patients with fractures from fragility are not aware of muscle weakness, since these patients fall more often, have more previous fractures, and present lower scores for instrumental activities of daily living. (Tables 2 and 3) To explore this fact, a future prospective study in this group of patients will objectively explore muscle strength.

Lower vitamin D intake among the OF group in relation to the OA group associated with more previous fractures may indicate a failure in primary and secondary prevention of osteoporotic fractures. As mentioned, the OA group was monitored by a multidisciplinary team for OA and comorbidities.^9^ ^,^ ^18^

Secondary osteoporosis occurs when an underlying illness, disability, or drug causes osteoporosis. We failed to ask specifically about hyperthyroidism, and found that the OF group showed less hypothyroidism than the AO group, indirectly corroborating the fact that hyperthyroidism tends to be more frequently associated with osteoporosis, among the endocrine diseases.^20^

Considering the surgical treatment that the hip fracture requires, in this tertiary center we found a much greater number of hip fractures than other fractures caused by osteoporosis (spine, wrist, and shoulder). (Table 4) However, the patients had densitometric osteoporosis and most did not take calcium replacement, vitamin D, or medication for osteoporosis, (Tables 2 and 4) showing the need for an educational program and multidisciplinary treatment for these patients which takes into account the financial, physical, and psychosocial problems that affect the individual, family, and community.^1^

## CONCLUSIONS

Together, race, marital status, previous falls, foot pathologies, previous fractures, and IADL scores define the profile of patients with osteoporotic fractures in this center.
